# Progress in the Identification of Dengue Virus Entry/Fusion Inhibitors

**DOI:** 10.1155/2014/825039

**Published:** 2014-07-24

**Authors:** Carolina De La Guardia, Ricardo Lleonart

**Affiliations:** ^1^Center for Cellular and Molecular Biology of Diseases, Institute for Scientific Research and Technology Services (INDICASAT-AIP), P.O. Box 0843-01103, City of Panama, Panama; ^2^Department of Biotechnology, Acharya Nagarjuna University, Nagarjuna Nagar, Andhra Pradesh 522 510, India

## Abstract

Dengue fever, a reemerging disease, is putting nearly 2.5 billion people at risk worldwide. The number of infections and the geographic extension of dengue fever infection have increased in the past decade. The disease is caused by the dengue virus, a flavivirus that uses mosquitos *Aedes* sp. as vectors. The disease has several clinical manifestations, from the mild cold-like illness to the more serious hemorrhagic dengue fever and dengue shock syndrome. Currently, there is no approved drug for the treatment of dengue disease or an effective vaccine to fight the virus. Therefore, the search for antivirals against dengue virus is an active field of research. As new possible receptors and biological pathways of the virus biology are discovered, new strategies are being undertaken to identify possible antiviral molecules. Several groups of researchers have targeted the initial step in the infection as a potential approach to interfere with the virus. The viral entry process is mediated by viral proteins and cellular receptor molecules that end up in the endocytosis of the virion, the fusion of both membranes, and the release of viral RNA in the cytoplasm. This review provides an overview of the targets and progress that has been made in the quest for dengue virus entry inhibitors.

## 1. Introduction

Dengue fever, dengue hemorrhagic fever, and dengue shock syndrome are caused by the dengue virus. According to the World Health Organization (WHO), dengue is one the most common mosquito borne diseases in the world [1]. It is estimated that up to 3.6 billion people live at risk of getting the disease [2]. Dengue virus is transmitted to humans by infected* Aedes* mosquitoes,* A. aegypti and A. albopictus,* which are distributed in tropical and subtropical areas and are widespread in urban and rural areas [1]. At present,* A. albopictus* can be found in temperate countries [3].

There is some uncertainty in the number and distribution of dengue cases due to the lack of reliable information and misdiagnosis and/or misreporting, thus emphasizing the importance of compiling more extensive records on dengue transmission [4]. It is estimated that dengue fever is present in 128 countries, including all continents, with figures differing from those reported by CDC and WHO [5, 6]. A recent study estimated that in 2010 there were 96 million apparent and 294 million unapparent dengue infections worldwide, with more infections in Asia (70%), followed by the Americas (14%) and Africa (16%) [7].

The pathogenic flaviviruses primarily include the four dengue serotypes, the Yellow fever virus, the West Nile virus, the Tick borne encephalitis virus, the Murray valley encephalitis virus, and the Japanese encephalitis virus. Dengue virus has an icosahedral symmetry, with diameter between 500 Å (mature virion) and 600 Å (spiky immature virion) [8]. The virus genome consists of a single stranded, positive, 11 Kb RNA coding for a single polyprotein. The polyprotein is cleaved in the cytoplasm into several structural and nonstructural polypeptides [9].

The structural proteins include the capsid (C), premembrane (PrM)/membrane (M), and envelope glycoprotein (E) that contains three main domains. These proteins are involved in the formation of the viral particle. The dengue virus membrane M protein has three portions, an extended N-terminal loop, an amphipatic perimembrane helix, and a pair of transmembrane helices [10]. The capsid protein of dengue consists of a dimer with four helices [11]. Ma et al. proposed that these helices may interact with the viral membrane or with viral RNA [12]. The nonstructural proteins (NS1, NS2a, NS2b, NS3, NS4a, NS4b, and NS5) are responsible for the viral replication, assembly, and immune response escape [9].

**Figure 1:**
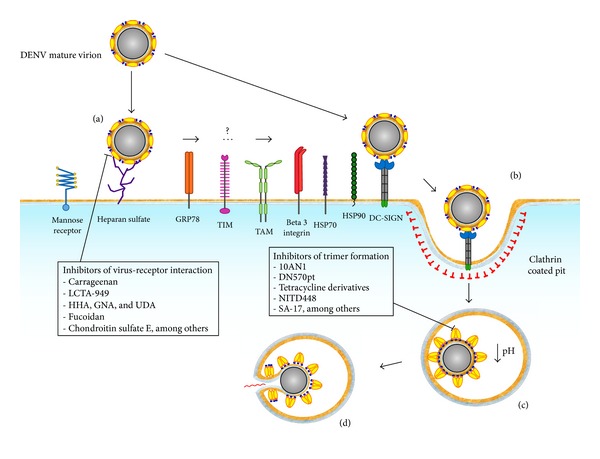
Schematic representation of the dengue virus entry process and possible antiviral targets. The dengue virus makes use of cellular membrane receptors and attachment factors to find its way to the cytoplasm. First, the mature virion gets attached to a cellular membrane receptor (a). It is not clear whether single interactions or sequential usage of several molecules is required to trigger the endocytic, clathrin-dependent pathway (b). The endocytic vesicle becomes a late endosome (c), where acidification triggers conformational changes on the E protein dimers to become fusogenic trimers. Finally, pores are formed and the genome of the virus is released into the cytoplasm (d). Possible antiviral targets are shown with examples of compounds inhibiting the step.

The entry of the dengue virus into the host cell is a complex process, mediated mainly by E glycoprotein. The first step of dengue virus entry is the binding of the viral E glycoprotein to a cellular receptor and/or attachment factors (Figure 1). Several of these receptor/attachment factors have been identified and are considered important targets for the development of antivirals, as explained in detail later in the text. Following receptor binding, the virus gets internalized via clathrin-dependent endocytosis (reviewed in [13]).

The dengue virus entry pathway is highly dependent on the cell type and viral strain. Although evidence suggests that the main way of entry for dengue virions is receptor initiated-clathrin mediated endocytosis [14], direct fusion through the plasmatic membrane has also been observed in particular cell lines [15, 16]. There is evidence that DENV-2 is able to use a clathrin-independent, noncaveolar, dynamin sensitive endocytic route which is also independent of macropinocytosis or phagocytosis [14]. The same virus, however, employs a classical clathrin-dependent pathway to enter human A549 cells. The “classical” postendocytosis fusion pathway depends on acidic pH to induce a conformational change in the viral envelope (E) protein to expose and insert the fusion loop into the endosomal membrane [17–19]. Once the endocytic and the viral membranes are bridged by the rearranged E glycoprotein, a final folding back movement of domain III brings the membrane closer to sequentially form apposing nipples, hemifusion stalk, and finally fusion pores. As a result, the membranes fuse and the viral RNA enters the cytoplasm [17].

Two broad directions have been undertaken to fight this disease. Most community level efforts are focused on reducing the mosquito populations, while at the patient level researchers are trying to develop vaccines and antivirals. The fight to reduce mosquito densities has been partially successful in some places, but the rapid development of resistance to chemicals and many other operative hurdles hamper the efficacy of these programs [20]. More sophisticated researches are trying to target the size of the mosquito population by releasing transgenic males carrying a dominant repressible lethal gene [21–23], but this solution might still have to overcome existing regulatory issues to be broadly applied.

The ideal solution for fighting dengue virus infection would be by developing a prophylactic vaccine. However, the fact is that current candidates have failed to give protection against all four serotypes, emphasizing the notion that a therapeutic agent—an antiviral drug—may be a key tool to fight the disease. An effective vaccine must protect against all four serotypes; otherwise, vaccinated individuals might be at risk of developing the severe form of dengue due to the phenomenon known as antibody-dependent enhancement (ADE). In this scenario, nonneutralizing antibodies opsonize the virions and mediate augmented entry and infection of cells expressing the Fc gamma receptor [24, 25]. This enhanced infection provokes proliferation of T cells, massive production of proinflammatory cytokines, and ultimately life-threatening systemic effects in infected patients [26–28].

Currently, there is no approved antiviral drug against the dengue virus. Several research groups have developed different approaches to identify molecules that inhibit dengue virus infection by targeting several structural and nonstructural viral proteins and its cellular receptors. One attractive approach could be blocking the virus before it enters the target cell. In this scenario, nature provides a proof of concept, as dengue virus generally induces the production of highly effective neutralizing antibodies in convalescent patients. The putative antiviral molecules targeting entry would not need to enter the cell, thereby relaxing structural and other constraints in the search and design of these molecules. Blocking the virus before entry could also reduce the hyperactivation of the immune system produced during the triggering of ADE. Dengue virus tropism in humans involves cells from liver and immune and endothelial systems. Other affected organs are lungs and brain (reviewed by [29]). Permissive cells include Langerhans cells, keratinocytes, monocytes, macrophages, and leukocytes. Also, bone marrow stromal cells are susceptible for dengue virus.

Several approved antiviral drugs show that entry inhibition is a feasible strategy to fight viruses. The anti-HIV drug maraviroc binds the virus coreceptor CCR5 [30], while enfuvirtide is able to block the viral protein GP41, reducing the fusion between viral and cellular membranes [31]. The drug Myrcludex-B, a lipopeptide based on the sequence of the preS1 domain of the HBV envelope protein, currently in clinical trials, is capable of stopping* de novo* viral infections [32]. This review provides an overview of the progress achieved in the search of dengue virus entry inhibitors, by targeting key molecules involved during this process.

## 2. Envelope Glycoprotein Structure Overview

The E glycoprotein is a class II fusion protein, mainly composed of *β* sheets and a short transmembrane domain for insertion into the viral membrane. This protein is highly flexible, undergoing significant conformational changes during the maturation and the fusogenic stages of the viral life cycle [17, 18, 33, 34]. In the mature virion, the E glycoprotein adopts a polygonal conformation, where dimers are arranged in polygons covering the surface of the viral particle [35].

Flavivirus virions structural studies show that the E glycoprotein is formed by three domains, in addition to a membrane proximal stem and a transmembrane anchor [17, 34, 36]. A central domain, the domain I, is formed by eight *β*-strand barrels containing two insertion loops. The dimerization domain, domain II, contains hydrophobic sequences that are conserved among all flaviviruses. These hydrophobic sequences, also known as fusion peptides, are responsible for the insertion of the rearranged E trimer into the cellular membrane during fusion [17, 33, 36–38]. Domain III is an immunoglobulin-like carboxy terminal domain, responsible for the initial cellular receptor binding [39]. The structure of this domain is arranged in a *β* barrel configuration, connecting with domain I [17].

Structural studies using X-ray crystallography and NMR solution structures of the domain III have shown that DENV-3 and DENV-4 contain nine *β*-strands, while DENV-2 has an extra *β*-strand [36, 40, 41]. The domain III can act as a virus fingerprint, due to the primary structure difference within the four dengue virus serotypes [42]. While the domain I and domain II are connected through four polypeptide strands, the domain I and domain III are connected by only one strand, working as a molecular hinge [18, 34]. The DI/DIII linker is a flexible region, allowing the interdomain rearrangement of E glycoprotein during the prefusion stage [43]. Several amino acids within this region have been identified as key for the viral replication and fusion [44, 45].

The E protein stem region is located at the C-terminus of the protein, from residues 395 to 450, and it is part of an outer lipid leaflet of the viral membrane [35]. This region consists of two *α*-helices (EH1 and EH2) connected by a conserved sequence [18, 38]. The EH1 and EH2 helices are amphipathic and slightly positively charged [35]. The arrangement of these helices differs depending on whether the virion is mature or immature [18]. The stem is important during the entry/fusion process because it promotes the E trimer assembly within the endosome [38].

The hydrophobic pocket is located in the domain I-domain II interface. This region plays an important role in E glycoprotein rearrangement, acting as a hinge that allows the exposure of the fusion peptide located in the domain II [34, 36, 46]. It has been proposed that the shift of two *β*-strands (*β*-hairpins) located at the hydrophobic pocket is the key structural element for the initiation of the E glycoprotein rearrangements, induced by a low pH environment in endosomes [36]. The hydrophobic pocket has been extensively used as a target for molecular docking approaches in order to identify molecules able to bind and ultimately show antiviral activity.

The E glycoprotein secondary and tertiary structure suffers significant rearrangements in order to adopt the fusogenic conformation [33]. This conformational change is triggered when pH is lowered inside the late endosome (Figure 1). These structural changes are involved in the dissociation of dimeric E glycoprotein and the formation of homotrimers, which point away from the virion surface and expose the fusion loops ready to be inserted into the endosomal membrane [19, 47–49]. These rearrangements are possible due to the linkers between domains [17]. During these changes, the domain II rotates approximately 30° with respect to domain I [17]. The hydrophobic core beneath this hairpin acts as a hinge, allowing the rotation between the domain I and domain II [17]. Domain III undergoes a significant rotation of about 70° toward domain II [17].

Dengue virus E glycoprotein adopts two main conformations: dimeric in the mature virion and trimeric in the immature virion and during the adoption of the fusogenic conformation inside the endosome [17, 33, 34, 36]. In the dimeric conformation, the antiparallel dimers lay flat on the virions surface, while trimers point away from the viral membrane with a conical rod shape to give the virion a spiky appearance. The fusion loop, which is exposed at the tip, contains three highly conserved amino acid residues, Trp101, Leu107, and Phe108 [17, 18, 33]. While the dimeric conformation is reversible, the transition to the trimeric conformation is irreversible.

The conformational changes leading to the formation of E glycoprotein trimer are thought to be initiated when low pH in the endosome is sensed by five histidine residues which are conserved among flaviviruses. These histidines are distributed in the three domains of the protein and in the stem region [10, 33, 50, 51]. Histidine 323, located at the DI–DIII interface, functions as a pH sensor and also stabilizes the trimer postfusion conformation. This stabilization might be due to the presence of a salt bridge between His323 and Glu373 [50]. When His323 is protonated, the salt bridge dissociates and destabilizes the DI–DIII interphase [33, 50]. Other two widely conserved histidines, His209 and His7, located in the E glycoprotein and the M protein, respectively, may also work as pH sensors. Hydrophobic interactions between E glycoprotein and M stabilize the E glycoprotein and are important for the conformational changes. When these histidines are in their protonated forms, the E glycoprotein dissociates from M protein, allowing the formation of the E trimer [10].

Other important structural characteristics of the E glycoprotein of DENV-2, identified by X-ray crystallography and cryo-EM, are the glycosylated Asn153 in domain I and Asn67 in domain II [10, 36]. The Asn153 residue is conserved among flaviviruses and interacts with the dimeric form of the protein, helping in the dengue virus's infectivity, whereas Asn67 is only found in dengue virus strains and it is thought that this glycosylation is important for the dengue virus assembly or exit [18, 52].

## 3. Possible Receptors for Dengue Virus

The most important target molecules on the cellular side of the dengue virus entry process are the attachment factors and receptors (Figure 1). Several molecules have been identified as possible receptors for the virus in mammalian cells, including the dendritic cell-specific intercellular adhesion molecule 3-grabbing nonintegrin (DC-SIGN; CD209) [53, 54], the heparan sulfate [39], the CD14 [55], the mannose receptor [56], the HSP90/HSP70 [57], the glucose regulated protein 78 (GRP78) [58], the laminin receptor [59], and the TIM and TAM proteins [60]. There is experimental evidence suggesting that dengue virus may also enter human cells by interacting with other molecules, including the vitronectin receptor [61], the scavenger receptor class B type I [62], the claudin 1 [63, 64], and the natural killer cell activating receptor [65]. Several of these receptors have been considered potential targets for the development of antivirals against the dengue virus.

It has been suggested that binding of the virus to the cell may require multiple, sequential interaction with several types of receptors. DC-SIGN and glycosaminoglycans (GAGs) are the first line of attachment factors (Figure 1). A second line of higher affinity receptors may then be recruited to allow viral entry, providing a possible explanation for the diverse tissue tropism of the virus [66].

### 3.1. DC-SIGN

The DC-SIGN is a type II transmembrane protein that is abundantly expressed in immature dendritic cells. It is a C-type lectin with an extracellular domain that binds mannose containing carbohydrates with high affinity. DC-SIGN is involved in dendritic cell migration, T-cell priming, and antigen recognition and presentation [67]. DC-SIGN also efficiently binds diverse viruses such as HIV-1 [68], Ebola [69], and CMV [70]. The DC-SIGN molecule was also identified as a cellular factor required for productive infection of immature dendritic cells by dengue virus [53, 54]. This interaction appears to be through the high mannose N-glycan groups located at the E glycoprotein [71]. Although this protein is glycosylated in two sites, Asn67 and Asn153, the former is believed to bind DC-SIGN [52].

The interaction DC-SIGN-DENV has been proposed as an attractive target to tackle the infection [72]. A recent study showed that the main interaction between E glycoprotein and DC-SIGN occurs by the formation of hydrogen bonds between the mannose molecule attached to Asn67 and Asn272 of DC-SIGN and six salt bridges present in different amino acid residues of both molecules. Blocking the E glycoprotein glycosylation site Asn67 and the conserved residues at the DC-SIGN-DENV interface might impair infectivity of all dengue serotypes [72]. Recently, it was proposed that the inhibition of the initial interaction between dengue virus and dendritic cells could prevent a deleterious immune response [73].

### 3.2. Glycosaminoglycans

Heparan sulfate (HS) belongs to the family of glycosaminoglycans (GAGs), which are very abundant in cell membrane proteoglycans. HS is composed of chains of disaccharides with uronic or L-iduronic acids and an O-sulfated glucosamine derivative [74]. It is well known that several virus families use the GAGs to enter the target cells. It is also known that HS is involved in the first interaction with the DENV, probably through GAG-binding sites on E glycoprotein [75–78]. These GAG-binding sites are located in the domain III of the protein [75]. Positively charged residues on E glycoprotein bind to the negatively charged HS [39, 75]. Recently, it was demonstrated that two conserved residues, Lys291 and Lys295, are critical for the interactions of GAG–E glycoprotein [45].

### 3.3. Other Possible Receptors

Besides DC-SIGN, dendritic cells (DCs) also express Fc gamma receptors (Fc*γ*Rs). Evidence suggests that DENV uses two different ways to enter DCs, a primary infection through DC-SIGN into immature DCs and a secondary infection through Fc*γ*Rs in mature DCs [79]. Although DENV particles should be previously opsonized with nonneutralizing antibodies to follow the Fc*γ*Rs entry pathway, some authors consider these cellular molecules as receptors for the virus.

Recently, the TIM and TAM receptors were identified as new DENV cellular attachment molecules [60]. These proteins are involved in the engulfment and removal of apoptotic cells, recognizing the apoptotic marker phosphatidylserine (PtdSer). Since the viral membrane exposes PtdSer, the virus is able to enter cells by direct binding of TIM receptor or indirectly by the TAM receptor through the PtdSer binder molecule Gas6. TAM receptor activation may play an important role in dengue pathogenesis as it shuts down the expression of interferon pathway genes, a key cellular antiviral response [13, 80].

## 4. Targeting Envelope (E) Glycoprotein

The dengue E glycoprotein is by far the most important molecule during the viral entry process as it appears to be responsible for receptor recognition and attachment to the cell, the clathrin mediated endocytosis, and the subsequent fusion of viral and cellular membranes. This protein is able to interact with many diverse cellular molecules; thus, it is considered an ideal target to develop antivirals [33, 46, 81, 82] (Figure 1). Several parts of the dengue virus E glycoprotein might be suitable as drug targets, including the stem domain, the hydrophobic pocket, and the receptor binding domain III. Blocking any of these particular regions may interfere with the entry/fusion process of dengue virus (blocking compounds summarized in Table 1).

### 4.1. The Stem Domain

Recent studies confirmed the viability of the stem region as an inhibition target. The disruption of the secondary structure of EH1 and EH2 by site-directed mutagenesis affected the viral entry, suggesting a key role of the stem region in the initial step of dengue infection [83]. Using the Wimley-White interfacial hydrophobicity (WWIH) scales algorithm, five possible inhibition sites of DENV were predicted. When peptides were designed, synthesized, and tested for antiviral activity, the best performing candidate corresponded to the stem region [84]. The peptide DN59, corresponding to amino acids 414 to 444, showed an IC_50_ of 2–5 *μ*M against DENV-2 in LLC-MK2 cells. It was later found that this peptide is effective against all dengue serotypes through a virucidal mechanism. The peptide DN59 is able to destabilize the viral membranes causing the release of RNA and production of empty virions [85].

Peptides based on the sequences of the stem region (residues 419 to 447) of all dengue serotypes showed cross-reactive antiviral activity (IC_90_: 0.1 *μ*M–>6 *μ*M), although similar peptides from other flaviviruses are not effective against dengue viruses [86]. Notably, the highest inhibition levels on the dengue serotypes tested were not exerted by the homologous peptides. These authors found that the best correlate of inhibition related to the stem region was the hydrophobicity of residues 441–447. The peptides corresponding to the stem region are able to bind soluble E glycoprotein and block the viral fusion by binding a conformational intermediate of the envelope protein. These peptides are also able to bind mature virions, being transported into the endosome where they inhibit the fusion [86]. The same authors developed a screening system to look for small molecules able to interfere with the binding of stem based peptides and the E trimer [87]. Compound 1662G07 was identified as promising hit and several analogs were further developed and characterized (best IC_50_: 8 *μ*M).

### 4.2. Hydrophobic Pocket

Several tetracycline derivatives have shown affinity for the hydrophobic pocket. These molecules were identified using GEMDOCK, a tool used to predict potential ligands for the active site of a target protein. The compounds doxycycline and rolitetracycline showed a significant* in vitro* inhibitory activity (IC_50_: 55 *μ*M and 67 *μ*M, resp.) against dengue virus type 2 in BHK-21 cells [88]. The binding of these molecules to the hydrophobic pocket halts the rearrangements of E glycoprotein domains I and II during pH induced rearrangement.

The compound SA-17, a derivative of the anthracycline antibiotic doxorubicin, showed antiviral activity against dengue virus serotypes 1, 2, and 3 (EC_50_: 12 *μ*M, 1.2 *μ*M, and 1.7 *μ*M, resp.). As suggested by docking simulations, the SA-17 molecule appears to bind to the hydrophobic pocket, interacting with amino acid residues crucial for the fusion of the membranes (Ala50, Tyr137, and Gln200) [89, 90].

Using high throughput docking screening for hydrophobic pocket ligands, a thiophene-pyrimidine molecule (“compound 6”) was also identified as a potential dengue virus entry inhibitor. After optimization, one compound was able to inhibit all dengue serotypes in BHK cells (range EC_50_: 0.068 to 0.49 *μ*M) and three additional flaviviruses [91]. However, testing in an* in vivo* dengue viremia animal model revealed that this compound precipitates in the gastrointestinal tract [91].

An* in silico* study using GLIDE docking software with a proprietary collection of compounds and natural products reported the finding of a molecule that potentially binds to the hydrophobic pocket [92]. The compound NITD448 effectively inhibited DENV-2 in BHK-21 cells (EC_50_: 9.8 *μ*M). This molecule showed suppression of the fusion of membranes mediated by E glycoprotein, most likely through interaction with Lys128 and Gln52.

The compound A5 was also identified by an* in silico* docking screening, using the hydrophobic pocket as target [93]. This molecule showed strong antiviral activity against dengue 2, West Nile virus, and Yellow Fever virus (IC_50_: 1.2 *μ*M, 3.8 *μ*M, and 1.6 *μ*M, resp.) and very low* in vitro* cytotoxicity (CC_50_: >100 *μ*M). This and other studies suggest that compounds with central thiazole rings may be critical to the observed antiviral activity targeting the hydrophobic pocket [94]. Using a four-stage computational high throughput screening (HTS) of three National Cancer Institute compound libraries, P02 was identified as a potential *β*-OG pocket binder. Additional experiments demonstrated that P02 binds E glycoprotein and has antiviral activity (IC_50_: 13 ± 3 *μ*M) [95].

A different approach for the* in silico* identification of potential drugs is the rational design of inhibitor molecules. Structural data from prefusion dengue virus E glycoprotein was used as a model for the design of new DENV entry peptide inhibitors [96]. The residue-specific all-atom probability discriminatory function (RAPDF) score was used for peptide design, allowing the identification of amino acid sequences that are likely to have structural and binding stability [96]. Two peptides were identified with this approach, 1OAN1 and DN57opt, with inhibitory activity against DENV-2 in LLC-MK2 cells (IC_50_: 7 *μ*M and 8 *μ*M, resp.). Both peptides were shown to bind E protein causing structural changes on the surface of DENV-2 virions.

### 4.3. Other Possible Targets on E Glycoprotein

E glycoprotein domain III was identified as a putative receptor binding domain that also stabilizes the E glycoprotein structure. Besides being the main target for neutralizing antibodies, several studies have shown that soluble domain III by itself may act as an antiviral through diverse mechanisms. Soluble domain III acts as a dominant negative inhibitor of class II fusion proteins, possibly by interacting with a core trimer intermediate and interfering with the folding back of the lipid bilayers [46]. Purified domain III from DENV-1 and DENV-2 inhibits entry of these serotypes into human HepG2 and mosquito C6/36 cells [97]. Also, a fusion protein containing maltose binding protein sequences, used as a solubility tag, and DENV-2 domain III showed antiviral activity in insect and human cells (IC_50_: 10 *μ*M and 13 *μ*M, resp.), probably due to competition for cellular receptors [45]. Further emphasizing the viability of domain III as a target, recent studies showed that small peptides designed from this region were active against DENV-2 in LLC-MK2 cells (IC_50_: 35 *μ*M) [98].

Similarly, cellular receptor molecules that interact with domain III may also have antiviral activity. One such molecule, the sulfated polysaccharide fucoidan, blocks DENV-2 virus entry into target cells by binding to E glycoprotein domain III and competing with the cellular receptors (IC_50_: 4.7 *μ*g/mL) [99]. The high molecular weight compound curdlan sulfate (CRDS) is a sulfated polysaccharide with branched *β*-d-(1→3) glucan backbone and piperidine-N-sulfonic acid groups. It was first identified as an inhibitor of HIV-1 entry and propagation [100]. Ichiyama and colleagues showed that CRDS binds E glycoprotein, probably near the fusion loop, and strongly and selectively inhibits DENV-2 in LLC-MK2 cells (EC_50_: 7 *μ*g/mL) [101]. DENV-2 and DENV-3 were effectively inhibited, while DENV-1 and DENV-4 required a much higher concentration of the compound. The authors proposed that CRDS interacts with residues of the kl hairpin, obstructing the shifting motion required for the dimer-trimer transition.

Some studies have shown that carbohydrate binding agents (CBAs) possess antiviral activities against DENV, because its entry into human target cells is carbohydrate-dependent (Table 1). CBAs also inhibit the DC-SIGN independent entry pathway of DENV in monocyte derived dendritic cells, interrupting the interaction between DENV and DC-SIGN [73]. It was reported that plant lectins, which are an important source of CBAs, are also able to interact with DENV E glycoprotein. Several plant derived CBAs, such as the lectins HHA (*Hippeastrum hybrid*), GNA (*Galanthus nivalis*), and UDA (*Urtica dioica*), showed strong antiviral activity against all serotypes of DENV (lowest EC_50_: 4.6 nM, 3.8 nM, and 0.29 *μ*M, resp.) [73]. Pradimicin-S, a small soluble nonpeptidic CBA, was also demonstrated to have an antiviral effect against DENV-2 in monocyte derived dendritic cells (EC_50_: 19 *μ*M) [73]. This molecule is attractive because it may have better bioavailability than other CBAs. Evidence showed that the CBAs act at an early step of infection, most likely by binding mannose bearing E glycoprotein and blocking viral attachment.

A recent study has shown that the GAGs heparin and chondroitin sulfate E inhibited DENV in BHK cells, unlike the parent compounds chondroitin sulfate A and C (lowest EC_50_: 0.3 *μ*g/mL) [81]. Heparin and chondroitin sulfate E inhibit the viral infection by directly interacting with E protein and blocking entry. On the other hand, it was found that sulfated polysaccharides isolated from different types of algae compete with dengue virus during interaction with target cell membrane components showing a significant antiviral activity for all DENV serotypes in Vero cells (IC_50_: 0.12–20 *μ*g/mL) [102]. These compounds included sulfated galactan, fucan, and xylomannan-containing fractions from red seaweed and were particularly effective against DENV-2.

Other heparan sulfate-like molecules also interfere with the entry and multiplication of DENV. Carrageenans are molecules formed by linear chains of alternate (1–3)-*β*-galactopyranose and (1–4)-*α*-D-galactopyranose. The iota-carrageenan shows an inhibition of DENV-2 multiplication in infected Vero cells (EC_50_: 0.4 *μ*g/mL) [78, 103], probably due to interference with the binding of virions to the HS receptor. Also, a sulfated K5 polysaccharide from* Escherichia coli* inhibits dengue infection in human dermal microvascular endothelial cells (EC_50_: 111 nM). This molecule interacts with E protein domain III, competing with heparan sulfate proteoglycans [104]. Lee et al. tested several HS-mimetics, suramin, pentosan polysulfate, and PI-88 showing relevant inhibition of virus attachment/entry against DENV, JEV, WNV, and MVEV. However, only PI-88 was active in an* in vivo* murine model of dengue viremia [105]. Two sulfated galactomannans extracted from the plants* M. scabrella* (BRS) and* L. leucocephala* (LLS) were found to be active against YFV and DENV-1, LLS being about ten times more active in C6/36 [106].

More recently, LCTA-949, an aglycon derivative molecule from the teicoplanin antibiotic, was found to possess antiviral activity against flaviviruses, including DENV-2 [90]. The antiviral activity of this molecule was evaluated using cytopathic effect (CPE) reduction assays, showing potent activity against several flaviviruses, including DENV-2 in Vero cells (EC_50_: 6.9 *μ*M). It was reported that this molecule targets an early stage of infections and was able to reduce antibody-dependent enhancement (ADE)* in vitro*. In fact,* in vitro* inhibition of ADE has also been reported for several antiviral compounds [82, 101, 107].

In a recent study, Lin et al. tested two hydrolysable tannins, chebulagic acid and punicalagin, as possible dengue virus inhibitors [108]. The two compounds showed antiviral activity against several viruses including DENV-2 in Vero cells (EC_50_: 13.1 and 7.8 *μ*M, resp.). It was suggested that these molecules can not only directly inactivate viral particles before attachment but also interfere with the entry process by binding the viral E glycoprotein and blocking interaction with GAGs.

The antiviral activity of sea grass derived natural product, zosteric acid, and several synthetic derivatives was tested by Rees and colleagues [110]. The derivative molecule CF 238 showed significant inhibition against all DENV serotypes in LLC-MK2 cells (range IC_50_: 14–47 *μ*M). Notably, although the compound interferes with an early step during entry, it seems also to enhance the binding of the virions to the cell surface.

The E glycoprotein is the natural target of the humoral response against the virus. As naturally elicited antibodies do not provide protection across different serotypes, other approaches have been tried such as monoclonal antibodies (mAbs) and engineered derivatives like antibody fragments as another strategy for the blocking of dengue virus entry. It has identified three main epitope regions within dengue virus E glycoprotein: A (located at domain II), B (domain III), and C (domain I). The domains A and B epitopes allowed the generation of monoclonal antibodies with higher biological activity against dengue virus. Antibodies that were reactive to domain A epitopes neutralize the virus and may block virus cell membrane fusion. The domain B epitopes elicited mAbs that were potent neutralizers of virus infectivity and blocked hemagglutination; however, they did not block virus mediated cell membrane fusion [111].

Most anti-DENV mAbs neutralize at least in part, by disrupting the virus ability to bind to mammalian cellular receptors. The mAb 1A1D-2 recognizes a partially hidden epitope (antigenic domain B, E domain III) and inhibits dengue virus attachment. The possible inhibition mechanisms could be due to altering the distances between the glycans present in the E glycoprotein and impairing the binding to the DC-SIGN receptor [112]. Domain III is the E glycoprotein domain that elicits the strongest inhibiting mAb [113]. Shrestha et al. have found that there are differences in the neutralizing activity and protective potential of mAbs generated against several DENV-1 genotypes. However, they were able to identify two mAbs able to strongly bind and neutralize all genotypes [114]. Although promising, the potential use of these antibodies as therapeutic agents must take into account the possibility of generating ADE due to suboptimal neutralization in some patients. To tackle this limitation, some authors have engineered the antibodies to have deletions in the Fc region, avoiding the binding to Fc*γ*R [115].

## 5. Targeting Other Viral and Cellular Factors

Compound ST-148 was identified by using a high throughput screening for molecules able to block dengue cytopathic effect [109]. Notably, the compound was very potent against all dengue serotypes (range EC_50_: 0.016–2.8 *μ*M) and against several other flaviviruses. The ST-149 binds to the virion capsid protein, being active also in a murine model of dengue viremia.

High levels of viremia correlate with severe dengue disease [116]; therefore, reducing uncontrolled virus production should be a priority of antiviral therapeutic interventions. Several research groups have looked into the possibility of using RNA interference (RNAi) technology to shut down key components of the entry machinery of the virus. In one study, silencing the expression of CD14 associated protein, clathrin heavy polypeptide, and dynamin 2 in human monocytes produced a significant reduction of infected cells and virus production [117]. Following the same logic of simultaneously inhibiting receptor- and clathrin-dependent entry pathways, this group also reported silencing the expression of GRP78, clathrin heavy polypeptide, and dynamin 2 in human hepatocytes [118]. These silenced genes allowed an important reduction of intra- and extracellular viral load and the number of infected cells. Although promising, the use of RNAi as therapeutic agent is still at the experimental stage, with many technical issues to resolve before becoming a real antiviral alternative. It is likely, for example, that silencing one or several host genes to reduce viral yield may also induce important off-target effects. Nevertheless, RNAi technology is still an invaluable analytical tool to understand the molecular biology of the virus and to identify new therapeutic targets.

## 6. Concluding Remarks

An extensive body of experimental evidence indicates that the DENV, while having a relatively simple structural organization, is able to exploit numerous molecules and pathways to infect cells and evade the immune response. Unfortunately, the virus is also able to cause severe, often deadly disease in a fraction of infected individuals. Therefore, the search for antiviral drugs is a top priority, as development of vaccines will also take some time to achieve a potent and balanced response against the four dengue serotypes.

Inhibiting the entry of the dengue virus to avoid the infection is an attractive approach to develop potent and specific antivirals. These molecules will exert their effects without having to enter the cells, thereby avoiding strict structural and chemical constraints. This kind of antiviral agents will have the added advantage of potentially limiting the immune system hyperactivation that leads to severe dengue.

Dengue viremia is acute during the first days of infection, decaying rapidly after 4-5 days [119]. Therefore, an antiviral drug could have a short early therapeutic window to fight the virus. However, as development of severe dengue is associated with uncontrolled viremia, the use of an antiviral may help stop further progression into this serious stage. To that purpose, early diagnosis may be crucial. Although an early diagnosis is not an easy task in routine clinical settings, considerable research has been done to incorporate early and robust biomarkers to the diagnosis, such as chemokines IP-10 and I-TAC expression in peripheral blood [120].

In general, antiviral development is focused on targeting viral components or cellular determinants of the infection. Targeting viral components has yielded the highest number of candidates, yet this strategy is prone to the rapid development of resistance. On the other hand, targeting cellular components reduces the possibility of developing resistance but may have a higher incidence of adverse effects. This approach may take into account cellular factors required for viral replication and maturation or pathogenic processes.

The growing body of knowledge about the biology of the virus tends to favor the approach of targeting the viral determinants of the entry process, instead of trying to block all possible cellular receptors and attachment molecules. Particularly promising as a target is the E glycoprotein because of its key role in viral attachment through receptor interaction and subsequent endosomal fusion. Several specific targets within the E glycoprotein are promising, including the stem domain, the hydrophobic pocket, and the domain III.

Another important challenge for the development of antivirals against DENV is the issue of bioavailability of the drugs. Particular drug families may have different chemical and biological properties that may dictate their fate in the organism. However, most of the molecules described here are small compounds in the proof of concept stage of research. Promising compounds may then soon move into preclinical studies where specific questions about* in vivo* efficacy and bioavailability are addressed.

Particularly useful for rapid development of antivirals are the animal models for viral diseases. In the past few years, there have been great efforts in the development of animal models for dengue virus infections. Currently, there are several dengue disease animal models. The AG129 mice deficient in type I and type II interferon receptors have been used to study dengue induced ADE (reviewed in [121]). This animal model was recently used to test the efficacy of an *α*-glucosides I and II inhibitor and an iminosugar as possible antiviral treatments [122, 123]. Another dengue animal model has been established by transplanting human CD34+ cells into NON/SCID (nonobese diabetic/severe combined immunodeficient) mice, showing clinical signs resembling human dengue fever disease [124, 125]. A recent investigation established a dengue animal model using a humanized NOD/SCID/interleukin 2 receptor gamma mouse strain. This animal model allows the evaluation human infection after the inoculation of dengue virus by infected mosquitoes [126]. However, the lack of an appropriate and convenient animal model for dengue infection is an important weakness for the rapid development and implementation of new antivirals [121].

## Figures and Tables

**Table 1:** Inhibitors of dengue virus entry.

Compound	Remarks	Cell line	Dengue virus serotype	IC_50_/EC_50_^#^	Reference
1OAN1	Fusion inhibitor, peptide based on E protein domain II hinge	LLCMK2	DENV-2	IC_50_: 7 ± 1 *μ*M^3^	[96]

DN57opt	Fusion inhibitor, peptide based on E glycoprotein DI/DII beta sheet connection	LLCMK2	DENV-2	IC_50_: 8 ± 1 *μ*M^3^	[96]

DN59	Fusion inhibitor, E glycoprotein stem and E trimer binder, peptide based on stem sequence	LLCMK2	DENV-2	IC_50_: 2–5 *μ*M^1^	[84, 85]

Compound 6	Fusion inhibitor, E glycoprotein hydrophobic pocket binder	A549/BHK21	All	EC_50_: 0.068–0.49 *μ*M^1∗^	[91]

Rolitetracycline	Fusion inhibitors, tetracycline derivative, and E glycoprotein hydrophobic pocket binder	BHK21	DENV-2	IC_50_: 67 *μ*M^2^	[88]

Doxycycline	Fusion inhibitors, tetracycline derivative, and E glycoprotein hydrophobic pocket binder	BHK21	DENV-2	IC_50_: 55 *μ*M^2^	[87]

NITD448	Fusion inhibitor, E glycoprotein hydrophobic pocket binder	BHK21	DENV-2	EC_50_: 9.8 *μ*M^3^ IC_50_: 6.8 *μ*M	[92]

A5	E glycoprotein hydrophobic pocket binder	Vero	DENV-2	IC_50_: 1.2 *μ*M^2^	[93]

1662G07 and derivatives	Fusion inhibitor, E glycoprotein stem, and E trimer binders	BHK21	DENV-2	IC_50_: 8 *μ*M^1^	[87]

SA-17	Derivative of doxorubicin, possible E glycoprotein hydrophobic pocket binder	Vero/BHK21	DENV-1 DENV-2 DENV-3	EC_50_: 12 *μ*M^4^ EC_50_: 1.2 *μ*M^4^ EC_50_: 1.7 *μ*M^4^	[89]

LCTA-949	Analogue of the antibiotic teicoplanin, entry inhibitor	Vero	DENV-2	EC_50_: 6.9 *μ*M^1^	[90]

ST-148	Inhibitor of capsid protein, effective in animal model	Vero	All	EC_50_: 0.016–2.8 *μ*M^4^	[109]

E 419-447 peptides	Stem derived sequence, trimer binder	BHK	DENV-2	IC_90_: 0.1–6 *μ*M^2^	[86]

E 380-389 peptides	Domain III derived sequence, attachment inhibitor	LLCMK2	DENV-2	IC_50_: 35 *μ*M^2^	[98]

P02	Binds to DENV hydrophobic pocket	BHK	YFV-IRES-Luc	IC_50_: 13 ± 3 *μ*M^8^	[95]

HHA, GNA, and UDA	Carbohydrate binding agent	Raji/DC-SIGN^+^	All	EC_50_: 4.6; 3.8; 0.29 nM^5^	[73]

Pradimicin-S	Carbohydrate binding agent	Dendritic cells	DENV-2	EC_50_: 11 *μ*M^5^	[73]

PI-88, suramin, and pentosan polysulfate	Heparan mimetic	BHK	DENV-2	EC_50_: 200 *μ*g/mL^9∗^	[105]

Fucoidan	Heparan mimetic	BHK21	DENV-2	IC_50_: 4.7 *μ*g/mL^3^	[99]

Sulfated galactomannan	Heparan mimetic	C6/36	DENV-1	EC_50_: 200 mg/L^10∗^	[106]

DL-galactan	Heparan mimetic	Vero	DENV-2	IC_50_: 0.9–1 *μ*g/mL^1^	[103]

Iota-carrageenan	Heparan mimetic	Vero	DENV-2	EC_50_: 0.4 *μ*g/mL^1^	[103]

Zosteric acid, CF-238	Heparan mimetic	LLCMK2	All	IC_50_: 14–47 *μ*M^3^	[110]

Curdlan sulfate	Heparan mimetic	LLCMK2	DENV-2	EC_50_: 7 *μ*g/mL^6^	[101]

Sulfated galactan, sulfated xylomannan	Heparan mimetic	Vero	All	IC_50_: 0.12–20 *μ*g/mL^1^	[102]

Sulfated K5 polysaccharide	Heparan mimetic	HMEC-1	DENV-2	EC_50_: 111 nM^9^	[104]

Chebulagic acid, punicalagin	Hydrolysable tannins	Vero	DENV-2	EC_50_: 13.1 and 7.8 *µ*M^7^	[108]

Chondroitin sulfate E	Heparan mimetic	BHK21	All	EC_50_: 0.3 *µ*g/mL^3^	[81]

^#^IC_50_/EC_50_: in antiviral assays, half maximal effective concentration (EC_50_) refers to the concentration of compound causing 50% reduction of virus replication in cell based assays. Half maximal inhibitory concentration (IC_50_) is used when virus inhibition is estimated from *in vitro* inhibition assays, such as the inhibition of a viral enzyme. Sometimes both EC_50_ and IC_50_ values are used loosely to describe the same antiviral activity. In this review, we are following the original nomenclature used by each cited paper.

^1^Viral plaque reduction assay.

^2^Plaque formation assay.

^3^Focus forming assay.

^4^Virus induced CPEs.

^5^RNA quantitation.

^6^Cell viability.

^7^Viral EGFP (enhanced green fluorescent protein) expression.

^8^Reduction of luciferase activity.

^9^Flow cytometry.

^10^Immunofluorescens.

∗Tested *in vivo* in animal model.
